# Interprofessional team-based learning (TBL): how do students engage?

**DOI:** 10.1186/s12909-020-02024-5

**Published:** 2020-04-19

**Authors:** Annette Burgess, Eszter Kalman, Inam Haq, Andrew Leaver, Chris Roberts, Jane Bleasel

**Affiliations:** 1 The University of Sydney School of Medicine, Education Office, Faculty of Medicine and Health, University of Sydney, Edward Ford Building A27, Sydney, NSW 2006 Australia; 2 The University of Sydney, Sydney Health Professional Education Network, Faculty of Medicine and Health, University of Sydney, Sydney, Australia; 3 Faculty of Medicine and Health, The University of Sydney, Sydney, 2006 Australia

**Keywords:** Interprofessional, Team-based learning, Collaboration

## Abstract

**Background:**

Although challenging to integrate within university curricula, evidence suggests that interprofessional education (IPE) positively impacts communication and teamwork skills in the workplace. The nature of Team-based learning (TBL) lends itself to interprofessional education, with the capacity to foster a culture of collaboration among health professional students. Our study was designed to pilot an interprofessional ‘back pain’ TBL module for physiotherapy and medical students, and to explore their experience of the TBL process, using the conceptual framework of ‘knowledge reconsolidation’ to discuss our finding.

**Methods:**

Three hundred and eleven students participated in the TBL session: 222/277 (80%) of Year 1 medical students and 89/89 (100%) of Year 2 physiotherapy students. Students completed one interprofessional Musculoskeletal Sciences TBL session on the topic of ‘back pain’. A questionnaire including closed and open-ended items, was distributed to students immediately following completion of the TBL session. Descriptive statistics were used to analyse the quantitative data. Thematic analysis was used to code and categorise qualitative data into themes. Pre-class quiz scores were compared between the groups using a one-way between groups Analysis of Variance (ANOVA) test with Tukeys Post Hoc test.

**Results:**

In total, 117/311 (38%) of participants completed the questionnaire. Both medicine and physiotherapy students appreciated the opportunity to learn about the curriculum of another healthcare discipline, and their scope of practice; gain multiple perspectives on a patient case from different disciplines; and recognised the importance of multidisciplinary teams in patient care. Students felt having an interprofessional team of facilitators who provided immediate feedback helped to consolidate student learning and promoted clinical reasoning. An analysis of variance revealed no difference between Physiotherapy and Medical students’ pre-class quiz scores.

**Conclusion:**

Our study demonstrated that the small group and task-focused characteristics of TBL provided a student-centred teaching strategy to support the achievement of interprofessional learning goals. Students valued their interactions with other students from a different professional degree, the opportunity to problem solve together, and learn different perspectives on a patient case. The pre-class quiz results demonstrate that both groups of students had a comparative level of prior knowledge to be able to work together on the in-class activities.

## Background

The World Health Organisation has stated that “interprofessional education and collaborative practice can play a significant role in mitigating many of the challenges faced by health systems around the world” [1]. Suggested benefits of integrated healthcare systems include greater co-ordination and continuity of patient care, increased patient satisfaction and collaborative decision making [1]. Increasingly, evidence suggests that interprofessional education (IPE) positively impacts on communication and teamwork skills in the workplace [2–4]. Despite these benefits, health education has been slow to adopt its integration within university curricula, largely because of difficulties in timetabling, learner-level matching, funding sources, preparation requirements and teaching resources [5]. Instead, health care students have traditionally been educated within their ‘silos’, with limited opportunities for interprofessional activities [5].

With our increasingly complex healthcare systems it is important to consider how students learn to work together across disciplines [6]. Team-based learning (TBL) has gained popularity in medical education within the last 20 years; in the last 10 years its use has expanded across the health professions [7–9], and currently there is an emerging trend to use TBLs as an interprofessional education platform [10]. Healthcare is an interdisciplinary field that is reliant on the sharing of knowledge from various professionals with unique expertise, to provide better patient care. The nature of TBL lends itself to interprofessional education, with the capacity to foster a culture of collegiality among health professional students. Additionally, TBL sessions provide an opportunity for the role modelling of interprofessional teamwork, with clinicians from various health professions and basic scientists working as an interdisciplinary team to educate students.

### ‘Knowledge reconsolidation’ as a conceptual framework

Theories underpinning learning and teaching practices offer lenses to analyse educational methods [11]. ‘Knowledge reconsolidation’ is a conceptual framework proposed by Schmidt et al. (2019), suggesting that there are four psychological mechanisms that enable knowledge reconsolidation during the TBL process [12]:

1. **‘retrieval practice’** – occurs during the IRAT, as students are encouraged to access knowledge they have previously learned.
2. **‘peer elaboration’** - occurs during the TRAT, as students have the opportunity to help each other understand difficult concepts.
3. **‘feedback’** - occurs following the TRAT, as students receive feedback and clarification of concepts.
4. **‘transfer of learning’** – occurs during the problem-solving activities, as students apply their knowledge to solve clinically relevant problems.

We applied the conceptual framework of *‘knowledge reconsolidation’*, as proposed by Schmidt et al. (2019) to reconceptualise and understand interprofessional learning within the TBL process [12].

### Local context

TBL was introduced to Sydney Medical School (SMS) in 2017, within a systems based 4 year graduate entry Doctor of Medicine program. Adopting a blended learning approach, TBL allows educators to provide students with resource effective, authentic experiences of working in teams to solve real life clinical problems [13].

In 2019, based on our previous TBL experience, as well as wider literature evidencing the effectiveness of TBL in health education, we sought to incorporate an interprofessional TBL within the Musculoskeletal sciences block of the Year 1 medical program, and Year 2 of the physiotherapy program. Key principles of our TBL design included: prescribed out-of-class preparation, pre-class individual tests, and in-class team tests, immediate feedback, and clinical problem-solving activities. This study reports on this single pilot interprofessional TBL session in Year 1 of a graduate entry medical program during the 2019 Musculoskeletal Sciences block, involving Year 1 medical students, and Year 2 physiotherapy students.

This study was designed to pilot an interprofessional ‘back pain’ TBL module for physiotherapy and medical students within the Faculty of Medicine and Health at The University of Sydney, Australia. We also explored participants’ perceptions of their experience of the interprofessional TBL process, using the conceptual framework of knowledge reconsolidation to discuss our findings.

## Methods

### Sampling and participants

In total 311 students participated in the TBL session. This included 222/277 (80%) of Year 1 medical students (Doctor of Medicine), and 89/89 (100%) of Year 2 physiotherapy students (Bachelor of Applied Science (Physiotherapy)). Students completed one interprofessional TBL session. At the time of the study, the students had no prior interprofessional experience within medicine and physiotherapy.

### Learning outcomes of the TBL session

The learning topic of backpain for the TBL session was selected to provide shared learning outcomes for both sets of students. The key learning outcomes were:

- Describe the anatomy and function of the vertebral column
- Apply the anatomy and physiology of the spinal cord and spinal nerves
- Describe the mechanisms of radicular pain

The problem was aligned with the biomedical science content of the medical curriculum, and the clinical day in hospital practice. For the Year 1 medical students, this was their first Musculoskeletal TBL, but the fourth TBL session for the academic year. For the Year 2 physiotherapy students, this was a single session that was embedded in a unit of study that included a six-week module on assessment and management of lower back pain in a primary care setting. The physiotherapy students had not experienced TBL previously.

### Structure of team-based learning

The TBL session was 2.5 h in duration. It was held outside of the students’ regular weekly schedule, at a time that would fit within the timetable of both the medicine and physiotherapy students.

#### Team formation

Medical students remained in their established TBL teams and classes (made up of approximately 11 teams per class) that had been previously allocated for the duration of the Year 1 academic year. The student teams consisted of four to six members, that were allocated with the intent to evenly distribute students based on gender, international status, and science background. Two or three physiotherapy students were randomly allocated to existing TBL teams for this single pilot interprofessional TBL session. Therefore, in this pilot TBL, there were six to eight students in each team, with 11 or 12 teams of students making up each TBL class. In total, four ‘TBL classes’ ran simultaneously.

### The process of team-based learning

#### Prior to class

##### Pre-class reading

Prior to class, all students were allocated compulsory readings and pre-recorded lectures to review.

##### Individual readiness assurance test (IRAT)

All students were required to complete an online quiz before attending class. The quiz consisted of 10 multiple choice questions (MCQ), with one single best answer for each question. The questions were aligned with the pre-class reading and pre-recorded lectures. Students were provided with a 10 min window to complete the quiz, and at completion, they were provided with their total score, but were not shown which questions they had answered correctly, nor the correct or incorrect responses to the questions.

#### In-class schedule

##### Team readiness assurance test (TRAT)

The same MCQ quiz was repeated by the students in their teams. The test was administered online, and students used one laptop per team, with the intent of promoting discussion to establish team consensus.

##### Immediate feedback and clarification from the facilitators

The correct answers were then released and explained, giving immediate feedback on team responses. The facilitators offered clarification, particularly where individuals or teams had experienced difficulty.

##### Clinical problem solving activities

Students worked in their teams on problem solving activities, using knowledge consolidated through the prior steps. There was opportunity within the immediate feedback session for students to initiate discussion and challenge answers.

### TBL facilitators

Each TBL class had an interprofessional team of facilitators including: one rheumatologist, one physiotherapist and one academic with basic science expertise in anatomy, physiology and/or pharmacology. The facilitators had been provided with prior training in TBL facilitation by either attending a 1 h face-to-face training session, or reading a TBL tutor guide and watching an online video. The objectives of the facilitator training session was to provide facilitators with an outline of the topic, and instructions on the teaching method of TBL, including elements such as how to prompt clinical reasoning through questioning. The instructional video demonstrating the steps of our TBL model can be found: https://www.youtube.com/watch?v=VstOyzeITf0&feature=youtu.be

### Data collection and analysis

#### Questionnaire

A modified version of a previously validated questionnaire regarding the IPE TBL experiences, was distributed to students immediately following completion of the TBL session [14]. The questionnaires included closed items (using a five point likert-scale, with 1 being ‘strongly disagree’, and 5 being ‘strongly agree’). The quality of team processes were measured using items adapted from a validated questionnaire designed by Thompson and colleagues (2009) [14]. Additionally, attitudes towards interprofessional learning were measured using items from the Readiness for Interprofessional Learning Scale (RIPLS) [15]. For example; “*Shared learning with other healthcare students as a student will help me to become a better team worker*”. Open-ended questions were also asked to gain a greater understanding of students’ experience. These included: What were the best features of this Team-based learning (TBL) session? What new knowledge, skills and values have you learned from participation in this TBL session? What were the most difficult features of this TBL session? and How could this TBL session be improved?

### Data analysis

#### Questionnaire

Descriptive statistics were used to analyse the quantitative data. Differences in medical and physiotherapy student responses were tested using a Multivariate Analysis of Variance. Thematic analysis was used to build an understanding of the students’ experience of the TBL session. A portion of the data was read by the first author and analysed to identify initial themes. Following negotiation of meaning with the second author, a coding framework was developed and applied to the full data set [16].

#### Test scores

IRAT and TRAT scores were compared between the groups using a one-way between groups Analysis of Variance (ANOVA) test with Tukeys Post Hoc test.

### Ethics approval

The University of Sydney Human Research Ethics Committee approved the study (project number: 2019/223), and written consent was obtained from participants through completion of the survey.

## Results

### Questionnaire

In total, 117/311 (38%) of participants completed the questionnaire, 75 medical students (41 male, 34 female) and 42 physiotherapy students (17 male, 25 female).

**Fig. 1:**
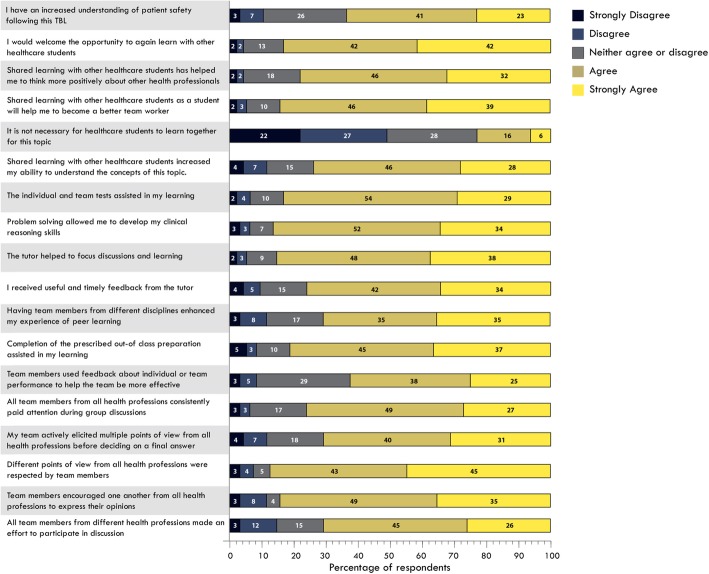
combined physiotherapy and medicine student responses to the questionnaire

**Fig. 2:**
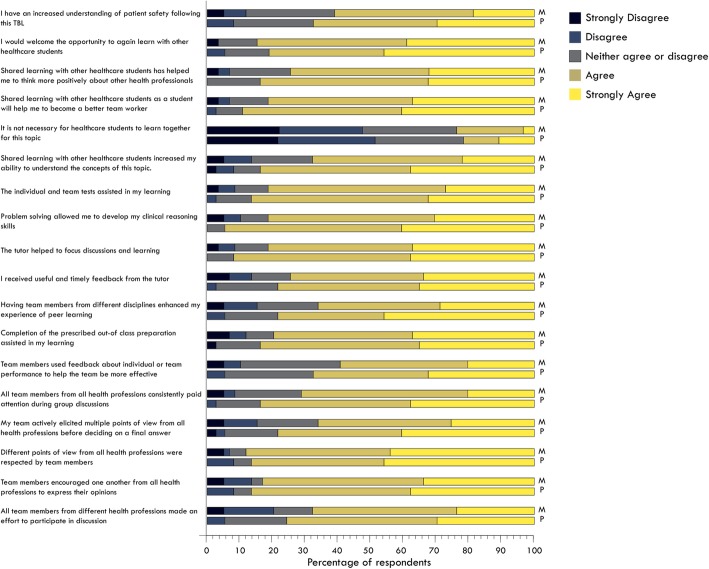
comparative physiotherapy and medicine student responses to the questionnaire

Student responses to closed items regarding their experience in the TBL are shown in Fig. 1. We note that we have collapsed agree and strongly agree when referring to the results shown in Fig. 1. Of note, there was no significant difference between Medical and Physiotherapy students’ perceptions of the TBL experience (Fig. 2). The preparation requirements were well received by students, with 82% of students agreeing “*Completion of the prescribed out-of-class preparation assisted in my learning”.* Students favoured the individual and team tests, with 83% agreeing that “*The individual and team tests assisted in my learning”*. Students were satisfied with the patient case, with 86% agreeing that “*Problem solving allowed me to develop my clinical reasoning skills*”. Feedback was well facilitated, with 76% of students agreeing that *“I received useful and timely feedback from the tutor”, and* 86% of students agreeing that *“the tutor helped to focus discussion and learning*”.

Notably, 88% of respondents agreed that *“Different points of view from all health professions were respected by team members”,* and 83% agreed that *“Team members encouraged one another from all health professions to express their opinions”.* Eighty four percent agreed that they would *“welcome the opportunity to again learn with other healthcare students*”. Additionally, 85% of students agreed that *“Shared learning with other healthcare students as a student will help me to become a better team worker”.* In considering areas for future improvement, only 71% of students agreed that “*all team members from different health professions made an effort to participate in discussion”.* The 64% agreement around understanding patient safety issues and the 63% concordance with the effective use of feedback in team performance again suggested a useful areas for optimisation.

### Responses to open-ended questions

Participant responses to open ended questions are displayed in Tables 1 and 2. Qualitative data is presented within themes in each of these tables. Table 1 presents students’ perceived “Best features of the TBL session” and “New knowledge, skills and values learned from participation in the TBL session”. In summary, students appreciated the opportunity to learn about the curriculum of another healthcare discipline, and their scope of practice; gain multiple perspectives on a patient case from different disciplines; and recognised the importance of multidisciplinary teams in patient care. Students felt having an interprofessional team of facilitators who provided immediate feedback, and brought their clinical experience to the class, helped to consolidate student learning and promoted clinical reasoning. Additionally, the physiotherapy students appreciated the opportunity to practice a different learning and teaching method (TBL).

**Table 1:** The best features of the Team-based learning (TBL) session and new knowledge, skills and valued learned from participation in the TBL session

**Opportunity to observe and practice difference learning and teaching pedagogies used in other disciplines** The physiotherapy students appreciated the opportunity to experience the structure and design of TBL, a relatively new pedagogy within health care education.	*Accessing different learning resources, instructors and styles from other disciplines to consolidate learning. The flipped lectures were particularly useful. (physiotherapy student)* *structure of the session..Open and interactive style of learning* *Discussion in groups + feedback in large group … Drawing and representing the key points visually – drawing mechanistic flow chart. (physiotherapy student)*
**The preparation material prior to class** Students appreciated the opportunity to learn content prior to attending class	*The preparation (especially the FLIPPED lectures) were really useful and well thought out; they had very clear messages for each one and linked strongly back to clinical relevance. (medical student)* *information from pre tute online lectures are useful, able to learn about clinical conditions from a different perspective (physiotherapy student)*
**The pre-class and in-class quizzes** Students found the IRAT helped in preparation, the TRAT immediately taken in class promoted collaboration in a new interprofessional team	*The pre-work information was very useful to help understand what would be discussed in the TBL … the pre-session quizzes. (physiotherapy student)* *The tRAT was useful to establish some level of collaborative discussion within the team. (medical student)*
**Knowledge of other curricula at an early stage, and appreciated the recognition of the multidisciplinary teams in patient management** Students found it beneficial to learn about what students from other disciplines were being taught	*Interdisciplinary work is very important as many chronic illnesses and disabilities require multi disciplinary involvement and management. I found it very beneficial as a physiotherapist student to learn that the medicine students were thinking about patient centred care as opposed to a biomedical model of intervention. (physiotherapy student)* *Good to have other members chat about their experiences and training programs …*. *Good exposure of their knowledge on the topic and their qualifications/trainings earlier on. (medical student)*
**Opportunities for collaboration** Student valued the chance to collaborate with students from other disciplines and consider different perspectives	*Talking with peers and negotiating. Engaging with other HCP forces you to see things from their perspective which is always a useful exercise (medical student)* *The ability to work with other health professionals* *Knowing the perspectives of different health professions regarding the same condition (physiotherapy student)*
**Differences in curricula** Student found the session allowed them to learning about the curriculum and topics taught in other disciplines, and their scope of practice	*Working with allied health personnel and understanding the scope of their practise …*. *It was great to learn from the physio students about their perspectives on back pain and how they have been learning this topic different to medicine* *(medical student)* *Discussion of the different approaches to management, learning a bit more about the details of what physiotherapists do to alleviate back pain (physiotherapy student)*
**Different perspectives** Students were able to gain multiple perspectives from different disciplines	*Different perspectives and approaches to learning, understanding and applying information helped* *us gain a wider knowledge of the topic. (medical student)* *It was very enriching to be able to discuss ideas and discover alternate ways of thinking when it comes to patient management and treatment plans (physiotherapy student)*
**Multidisciplinary team of TBL facilitators** Students found it beneficial to be taught by a multidisciplinary team of facilitators who brought their clinical experience	*The tutors were very helpful and interactive. Just fabulous* *Listening and learning from the insights from the multi-disciplinary panel of tutors, some of whom were* *doctors, physiotherapists and basic scientists. Applying what we learn in lectures to a common disease/condition in the community helps us to understand the principles of diagnosis and management, as well as emphasise their relevance. (physiotherapy student)* Tutors provided more well-rounded knowledge of the topic from different viewpoints … .. *Having physiotherapists attend the TBL along with clinicians to appreciate the different approaches and perspectives of the healthcare providers in diagnosing and managing musculoskeletal problem (medical student)*
**Facilitator feedback** Students appreciated the feedback provided by an interdisciplinary team of facilitators	*The ability to ask academics directly about questions - in lectures it is often intimidating … I liked that there were medical and physiotherapy tutors, they were all keen to offer helpful* *and multidimensional explanations of patient care. (physiotherapy student)* *The tutors for this TBL session were really helpful in giving us relevant information and advice about the different topics and being able to work with physiotherapy students enabled us to* *experience how the real world situation could potentially look like. (medical student)*
**Patient case** Both medical and physiotherapy students found the patient case relevant and well aligned with their curriculum	*The content was actually relevant to what we’re covering in lectures, practicals and clinical days (medical student)* *Interesting case: the history and examination information were succinct and relevant. The flow and components in the case study (physiotherapy student)*
**Clinical reasoning** Students valued the opportunity to apply clinical reasoning to an authentic clinical case	*Brilliant topic and clinical features discussed* *as clinical application … relevance to clinical context … lots of opportunity to develop clinical reasoning. (medical student)* *Applying what we learn in lectures to a common* *disease/condition in the community helps us to understand the principles of diagnosis and management as well as emphasise their relevance. (physiotherapy student)*
**Importance of teamwork in patient care** The session emphasised to students the role and importance of multi-disciplinary work	*The importance of teamwork in coming up with a coherent and effective clinical reasoning. Back pain is a multifaceted issue and treatment should be multimodal. (medical student)* *It helped me to better understand the role of teamwork among a number of healthcare disciplines when treating patients (physiotherapy student)*
**Roles of other health professions** Participation in the session helped to develop students’ knowledge and understanding about the roles of other health profession	*I now have a better understanding of the other roles and knowledge that other health care professional have. (physiotherapy student)* *Knowledge about physiotherapy practices.. I learnt a little about physiotherapists which was nice. Just about the practicalities of their work such as that referrals from doctors are negligible in terms of their clientele, they practically all present of their own volition. (medical student)*
**Preparation for practice** Students developed their understanding of what is taught in different healthcare courses	*What physios can offer and their qualifications …* *That Medicine students are learning very similar things to us (physiotherapy student)* *I gained perspective and respect for another health profession and what they were learning in their course, which is good practice for the future. (medical student)*
**Shared decision making** Students developed their appreciation of active listening and shared decision making	*I have learnt the importance of listening to my peers actively and also incorporating their ideas to come to a better decision or conclusion. (physiotherapy student)* *Keeping an open mind when encountering patient cases. There could be more than one condition bothering the patient (medical student)*

**Table 2:** The most difficult features of the TBL session and suggestions for improvement

**The uneven distribution of medicine and physiotherapy students in each team.**	*Had to actively explain rationale for physio intervention as there were less phsyio students in the group whereas medical student clinical reasoning was readily accepted by group (physiotherapy student)* *Perhaps physiotherapy students may have felt intimidated and outnumbered by the Medical students? This meant that we had to be very mindful to include them in discussion and explicitly asked for opinions – instead of them offering opinion. This may indicate they weren’t entirely comfortable? (medical student)*
**Introducing physiotherapy students into established medical student team provided difficulties**	*As the physio students hadn’t had a TBL before sometimes I felt our group sort of rushed through as we are familiar with the format which may have been confusing for the physio students. (medical student)* *I feel the interprofessional aspect would be more successful from day one TBL. Introducing two (physiotherapy) students into an existing group of five that already have a way of working together can make it harder for them to participate (medical student)*
**Medical students wanted to hear from physios about treatment options** Some students suggested that the class may have been more effective if information was specifically sort from each discipline	*I would also have liked to have the TBL maybe geared a little towards treatment options, since a lot of it was diagnosis, and the physio students could definitely have given a lot of insight on treatment that they didn’t get an opportunity to go into.* *(medical student)* *It was good to have physiotherapy students in our group, but their input into group discussions was somewhat limited … It may be helpful for the physiotherapy students to be given a bit more background into TBL sessions and/or have some specific questions targeting physiotherapy (*e.g. *what sort of exercises are beneficial in management of the condition), to encourage participation from the students.*
**Group dynamics** Development of group dynamics was more difficult because the physiotherapy students were added to the medical student groups, rather than being part of a team from the beginning of a teaching block Students would have like additional instruction from tutors to ensure physiotherapy students were better included in the team	*Maybe since there were added team members, it was a bit more difficult to communicate - the group dynamic changed from what we had previously. Not saying it was bad, but it took a while for all to get used to it. … Have some guidelines on how to incorporate new team members to already existing teams. It’s always awkward to try and navigate new team members unless they become regular. (medical student)* *Integrating physio students in our already existing TBL group. Some guidelines (very high level) should be touched upon by tutors to ensure folks consults the physio students more effectively (medical student)*
**More structured discussion** Some students indicated that by having a structured discussion, with some questions being answered by physiotherapy students, and some by medical students would be beneficial.	*Perhaps having more specific questions targeted at each specialty (*e.g. *for physiotherapists - what exercises are* *most beneficial to manage low back pain; or for doctors - when would they refer the patient to a physiotherapist* etc.*) - to help engage all members of the group (medical student)* *High level guideline of roles and ways to operate as a team (time to discuss actual role in real life - how would referrals happen in real life and what would each need to know or do? (medical student)*
**A more diverse mix of students** Students wanted to see a greater mix of students in each TBL team	*Invite other allied health professions to get different perspectives to come up with a diagnosis and treatment plan (medical student)* *Getting more different types of professional involved (physiotherapy student)*
**A greater number of interprofessional TBL sessions**	*More TBL session for medical students to familiarise with including physiotherapy and other health allied students (medical student)* *More would be great!*:*) (physiotherapy student)*
**An opportunity for physiotherapy students to better integrate into the TBL session**	*Perhaps to give more time for physiotherapy and medical students to introduce and get acquainted to each other to allow more extensive discussion during the TBL session (medical student)* *Allowing students in each group to come into the session with a similar level of acquaintance as the rest of the* *group members (medical student)*

Table 2 presents students’ perceived “most difficult features of the TBL, and suggestions for improvement”. Students highlighted that the uneven ratio of medical and physiotherapy students, and this being the physiotherapy students’ first introduction to TBL may have hindered their contributions. Some students suggested a more structured discussion of the patient case, with questions targeting each discipline, may help to engage all members of the teams. Additionally, students indicated that they would like other healthcare disciplines included in the TBLs, and a greater number of interprofessional TBLs.

### IRAT scores

**Fig. 3:**
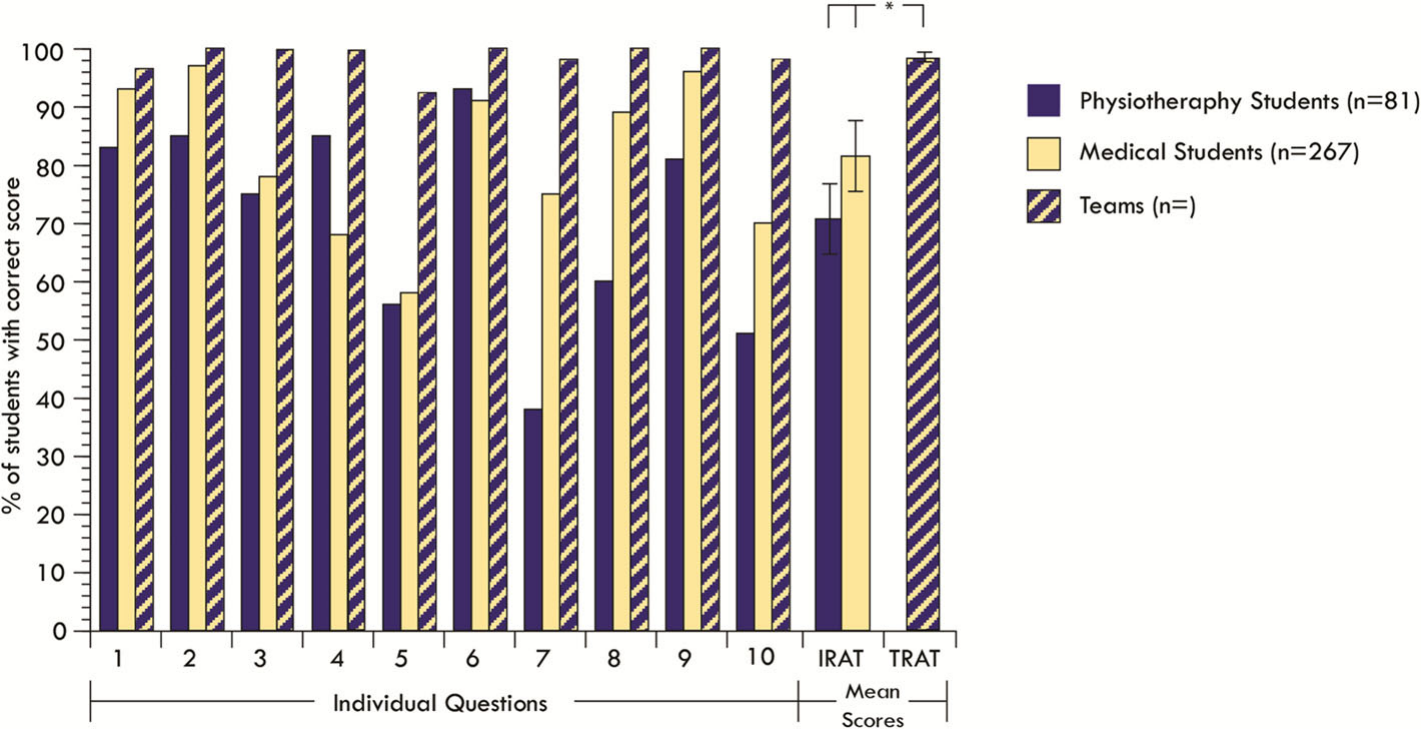
The percentage of students who were able to identify the correct answer for each of the IRAT and TRAT questions. (All teams outperformed the mean score of their individual team members)

The percentage of students who were able to identify the correct answer for each of the IRAT and TRAT questions is presented in Fig. 3. An analysis of variance revealed no difference between Physiotherapy and Medical students’ IRAT scores, suggesting that prior content knowledge of both disciplines was equivalent. Total TRAT scores were significantly higher than total IRAT scores (F (2, 27)=11.3, *p* = 0.000266), Post-hoc comparison using Tukey’s HSD revealed that this was true when medical (M = 81.5, *p* < 0.05) or physiotherapy students’(M = 70.7, *p* < 0.001) total IRAT scores were compared with the total TRAT (M = 98.5) scores of the interprofessional groups. While no comparison could be made at the level of the questions, it is worth noting that the total number of groups that identified the correct answer in the TRAT was always higher than individual student’s attempts during the IRAT.

## Discussion

This study sought to explore health professional students’ perceptions of their learning experience during a single interprofessional (physiotherapy and medicine) TBL session on the topic of ‘back pain’. Positive aspects included the ‘flipped classroom’ format, with pre-reading provided that was clinically relevant, the individual and the team test (IRATs and TRATs), with the provision of feedback. Students appreciated the opportunity to learn about the curriculum of another healthcare discipline, and their scope of practice; gain multiple perspectives on a patient case from different disciplines; and recognised the importance of multidisciplinary teams in patient care. Students felt having an interprofessional team of facilitators who brought their clinical experience, helped to consolidate student learning and promoted clinical reasoning. We use the themes of ‘retrieval practice’, ‘peer elaboration’, ‘feedback’, and ‘transfer of learning’ within the conceptual framework of ‘knowledge reconsolidation’ [12] to discuss our findings, and assist our understanding of students’ interprofessional learning within the TBL process.

### Retrieval practice (pre-reading and IRAT)

Occurring during the IRAT of TBL, retrieval practice encourages students to access new knowledge they have learned, which in turn helps to facilitate later retrieval of the same knowledge [12]. Known as the “testing phenomenon”, Glover (1998), demonstrated that students who take tests in between the initial learning of material, and their final examination achieve higher scores than those who do not [17]. Students’ scores from the IRAT indicate that physiotherapy and medicine students were equally prepared to participate in group discussions and problem-solving activities. In line with this, students felt that the pre-reading and the IRAT helped them to prepare and set them on a level playing field. This is inline with literature indicating that peer pressure of small group work encourages students to complete assigned study [18].

### Peer elaboration (TRAT)

Peer elaboration occurs during the TRAT, where students have the opportunity to help each other understand difficult concepts and gain a new perspective on knowledge [12]. Students commented that completing the TRAT in their small groups developed their appreciation for the multiple perspectives of another healthcare profession, and the similarities and differences in their respective curricula. They felt they learnt about the importance of actively listening to each other before reaching a decision on an answer. Recent literature suggests that early experience of IPE can enhance students’ readiness for further interprofessional learning and their attitudes to multidisciplinary teamwork [19, 20]. Students need to interact with each other to ensure that shared decision making is fostered, and team members listen to each other [4, 21] and the TBL format assisted this learning process. Students commented on the imbalance of medical to physiotherapy students in each team, and the late integration of physiotherapy students to established medical TBL teams. One of the principles of TBL, and in line with group dynamic literature [22, 23] is that teams need to work together over an extended period of time so that team dynamics develop, and communication becomes more open and collaborative, with trust and appreciation of diversity of knowledge [24, 25]. As reported in current literature, there are many logistical challenges of conducting interprofessional learning activities [26].

### Feedback (from facilitators following TRAT)

As well as testing, it is known that the learner benefits from the provision of feedback following testing [12]. The TBL format offered a feedback-rich learning environment. In each of the interprofessional TBLs, a rheumatologist, a physiotherapist and a basic scientist taught and provided feedback as a team. Team teaching involves educators working together to teach, while taking advantage of the particular competencies of each team member [27, 28]. Students commented that they were able to ask medical, physiotherapy and basic science facilitators questions in a learning format that promoted discussion. They felt the feedback and explanations provided by facilitators, and the different ‘styles’ and approaches to patient care helped to consolidate their learning. This aligns with other literature stating that the benefits of the team-teaching include students being provided with more than one explanation of a complex case; exposure to different teaching methods; more diverse interactions, debate and active discussion; and the role modelling of interprofessional collaboration [29, 30].

### Transfer of learning (clinical problem-solving activities)

The transfer of learning occurs when students apply what they have learned in one context to another [12]. This occurs during the problem solving activities of TBL. Our findings indicate the patient case and clinical problem-solving activities fostered students learning together, learning about each other’s roles in clinical practice, and highlighted the need for joint decision making in patient management. Literature suggests that the design of the clinical cases needs to ensure that the disciplinary knowledge of all team professions is fostered by the tasks within the problem solving activities [31, 32]. The case used (‘Back pain’) focused on diagnosis where there is overlap of medical and physiotherapy roles, particularly in an acute primary care setting, depending on who the patient chooses to see. Some students suggested a more structured discussion of the patient case, with questions targeting each discipline, may help to engage all members of the teams. The case could potentially be expanded to include management where the specific roles of each discipline is more distinct. For example, prescription of medication and advice about activity/exercise.

### Study limitations

The response rate 117/311 (38%) in this evaluative research is disappointing compared with other published reports. Bias may exist in that those who chose to respond may have favoured the TBL format, and our results may not be reflective of the views of the wider population. The qualitative analysis afforded by this mixed methods design gives a rich appreciation of the experiences of IPE in a particular context, but may not be generalizable to other settings. However, our data will be of relevance for those educators seeking to adapt the IPE TBL model described in their setting.

## Conclusions

Our study demonstrated that the small group and task-focused characteristics of TBL provides an opportunity to develop collegiality and collaboration among health professional students at an early stage in healthcare curricula. Team-based learning provided a student-centred teaching strategy to support the achievement of interprofessional learning outcomes. The interprofessional team-teaching strategy provided educators with the opportunity to role model collaborative teaching and facilitate the clinical application of theoretical knowledge. Students valued their interactions with other students from a different professional degree, the opportunity to problem solve together, and learn different perspectives on a patient case. While the test results demonstrate that both groups of students had a comparative level of prior knowledge, with good curricula alignment on the topic of back pain, the mismatch in the student cohort numbers compromised the TBL format. However, by overcoming our logistical challenges, we will embrace opportunities for interprrofessional TBLs across the health professions.

## Data Availability

Datasets supporting the conclusions of this article are included within the article. Additional data at the level of individual students is not available as per confidentiality agreements approved by the Human Research Ethics Committee, University of Sydney.

## Abbreviations

TBL: Team-based learning
PBL: Problem based learning
IRAT: Individual Readiness Assurance Test
TRAT: Team Readiness Assurance Test
SMS: Sydney Medical School
SMP: Sydney Medical Program
